# A Freak of Nature in an Entire Family of Children

**Published:** 1863-03

**Authors:** M. M. Eaton

**Affiliations:** Peoria


A FREAK OF NATURE IN AN ENTIRE FAMILY OF CHILDREN.

### AN IMPERFORATE OS UTERI.

By M. M. EATON, M. D., of Peoria.

April 23rd, 1862, was called with Dr. Hamilton, to go to Fond du Lac, in Tazewell County, to amputate a lot of supernumerary toes and fingers.

We found our friend with a family of three boys, all of whom had been born with six fingers on each hand, and six toes on each foot. Two of the boys had previously been relieved of their superfluous fingers. Six toes to a foot on all of them still remained, and caused the feet to be so wide that common shoes could not be worn, and made the feet look quite awkward. We administered Chloroform, and removed all the supernumerary toes and fingers, the wounds healed well, and left useful and well shapen feet and hands.

The father and mother were both well formed and healthy, and they told me that they never had known of any of their relatives having had any deformity of any kind.

Truly, there are many works of what is called nature, we, short-sighted mortals, cannot explain.

And this reminds me of the case of a married lady who consulted me last fall, on account of never having had the menstrual flow in her life. She said she had been married three years, was 19 years of age, lived several miles up the river in a small village; was a native of Ohio. Her mother, who came to the city with her to consult me, said she had never had her courses, and she wanted I should bring them on, if possible. I told her that I never prescribed for a case without knowing as much as possible relative to it, when there might be doubts whether she were well formed, I therefore, requested an examination; to which assent was given, adding that "she had been treated by several physicians in her place almost continuously, for the last four or five years, but no one had asked an examination." By examination, I ascertained that the breasts were undeveloped, and by examination found that the hair on the Mons Veneris was absent, and also, that her vagina was perfect. By the speculum, on most careful search, no opening into the womb could be found, although the womb was of usual size. I called in Dr. Frye, who verified my diagnosis. I told Mrs. H., although she was well formed (with this exception) I thought no operation was called for, and that the less medicine she took the better.

I believe in her case there is an absence of the Ovaries as well as an imperforate *Os Uteri;* otherwise, I should advise differently. A little reflection on this case will show how a false modesty on the part of a physician, or his patient, may sometimes be the means of great embarrassment and want of success with the former, and much trouble and unnecessary expense, and often positive injury to the latter.

